# Identifying Parabrachial Neurons Selectively Regulating Satiety for Highly Palatable Food in Mice

**DOI:** 10.1523/ENEURO.0252-19.2019

**Published:** 2019-11-19

**Authors:** Erica Rodriguez, David Ryu, Shengli Zhao, Bao-Xia Han, Fan Wang

**Affiliations:** Department of Neurobiology, Duke University Medical Center, Durham, NC 27708

**Keywords:** brainstem, Fos, novel tools, satiety

## Abstract

Food consumption is necessary for organisms to maintain metabolic homeostasis. Both extrinsic and intrinsic processes, relayed via intricate neural circuitry, orchestrate the initiation and termination of food intake. More specifically, there are functionally distinct neural circuits that mediate either homeostatic or hedonic suppression of feeding. Notably, being satiated is a positive feeling whereas food aversion is a negative feeling. While significant progress has been made toward elucidating neural circuitry underlying aversive appetite suppression in mice, the circuitry underlying homeostatic satiety is not fully understood. The lateral parabrachial nucleus (PB_L_) is known as a node that regulates various sensory and visceral processes. Here, we identified and selectively labeled neurons in the caudal lateral region of PB_L_ (PB_cl_) that are activated by consumption of condensed milk, chocolate Ensure, or peanut butter, which we refer to as PB_cl_-palatable-food activated neurons (PANs). Specific optogenetic activation of PANs induced positive place preference but decreased the consumption of high-caloric foods such as condensed milk, whereas silencing these cells significantly increased condensed milk consumption in feeding assays. Thus, the PB_cl_ PANs revealed here represent a novel neural substrate regulating caloric-sufficiency mediated satiation.

## Visual Abstract

**Figure:**
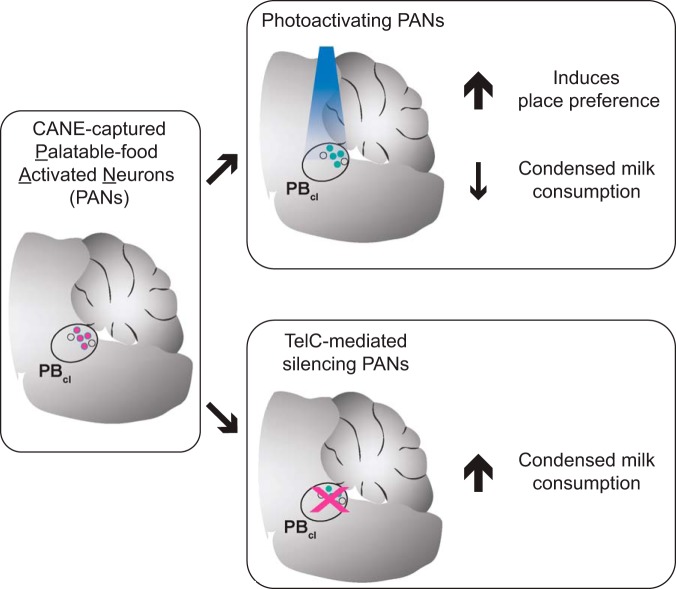

## Significance Statement

The lateral parabrachial nucleus (PB_L_) is critical for regulating somatic and visceral processes including thermoregulation, taste preference, thirst, and appetite. Recent work on satiety-promoting circuits has implicated the PB_L_ as an important node. Previous studies identified the PB_L_^CGRP^ population mediating aversive feeding suppression. However, the identity of the PB_L_ population mediating homeostatic satiety is poorly understood. PB_L_’s functional and molecular diversity makes it difficult to separate specific populations critical to regulating specific processes. We address the question by using activity-dependent capturing technology to molecularly identify and selectively activate and inhibit PB_L_ neurons activated by consumption of highly palatable food (PANs). We demonstrated that PANs are distinct from PB_L_^CGRP^ neurons and likely relay appetitive and caloric sufficient satiety signals.

## Introduction

Proper regulation of nutrient intake and food consumption is essential for survival and homeostasis in chemotrophic organisms. Feeding is heavily regulated by a multitude of signals coming from exteroceptive processes, such as the availability and taste of food, and interoceptive processes, such as feedback from the gut and adipose tissues (Chambers et al., 2013; Andermann and Lowell, 2017). The signals from these exteroceptive and interoceptive processes are relayed by multiple neural circuits that regulate the initiation and termination of feeding (Sternson and Eiselt, 2017). These neural circuits may undergo maladaptive plasticity in humans suffering from conditions such as anorexia nervosa and obesity, resulting in dysfunctional appetite (Farooqi and O’Rahilly, 2008). Additionally, within these neural circuits, there are a variety of subcircuits which regulate various aspects of feeding, such as hunger, palatable appetite, satiety, and aversive meal termination (Sternson et al., 2016; Sternson and Eiselt, 2017). This complexity makes it challenging to dissect which circuits regulate which aspects of appetite and satiety.

The termination of feeding behavior can result from two separate processes: either due to caloric sufficiency (feeling satiated) or due to receiving aversive anorexigenic signals such as malaise. Significant progress has been made toward the pathways underlying aversive feeding suppression. Specifically, several studies revealed a group of calcitonin gene-related peptide CGRP^+^ expressing neurons in the lateral parabrachial nucleus (PB_L_^CGRP^) as the key mediator of the aversive signal (Campos et al., 2016, 2018; Carter et al., 2013, 2015). Activation of PB_L_^CGRP^ neurons induces meal termination, anorexia, and affective nociceptive behavior (Carter et al., 2013; Cai et al., 2014; Han et al., 2015; Campos et al., 2016), whereas silencing these cells prevents anorexia in a cancer model (Campos et al., 2017). These PB_L_^CGRP^ cells receive both direct excitatory input from *Th^+^* and *Cck^+^* cells in the nucleus of the solitary tract (NTS; Kreisler et al., 2014; D’Agostino et al., 2016; Roman et al., 2016), as well as direct inhibitory input from agouti-related peptide (AGRP^+^) expressing neurons in the hypothalamic arcuate nucleus (ARC), which are hunger-activated and promote feeding (Aponte et al., 2011; Krashes et al., 2011; Atasoy et al., 2012; Betley et al., 2013; Burnett et al., 2016; Livneh et al., 2017). The PB_L_^CGRP^ neurons project to the central amygdala (CeA) and synapse with a subset of protein kinase c delta (*PKC-δ^+^*) expressing CeA GABAergic neurons to suppress feeding (Cai et al., 2014).

By contrast, our understanding of the circuitry underlying physiologic/homeostatic satiety-mediated meal termination remains incomplete. After food consumption, a cocktail of hormones and neurotransmitters is secreted from enteroendocrine cells residing in the stomach and small intestinal epithelium which signal caloric value and satiety. Vagal sensory afferents detect these signals and transmit the information to NTS (Grill and Hayes, 2009; Chambers et al., 2013; Gribble and Reimann, 2016; Williams et al., 2016; Roman et al., 2017; Kaelberer et al., 2018). Distinct groups of NTS neurons then send this information to multiple brain centers, such as the dorsomotor nucleus of the vagus, the PB_L_, ARC, and the paraventricular hypothalamic nucleus (PVH; Rinaman, 2010; Trapp and Cork, 2015; D’Agostino et al., 2016; Travagli and Anselmi, 2016). Interestingly, melanocortin-4 receptor neurons in PVH (PVH^MC4R^) send direct projections onto an unidentified population of neurons in the dorsal portion of PB_L_, which appears to be spatially distinct from where CGRP^+^ neurons are located in the ventral portion of PB_L_ (Shah et al., 2014; Garfield et al., 2015; Li et al., 2019). Activation of PVH^MC4R^ terminals in PB_L_ induced satiety, while silencing the projections increased food consumption in mice (Garfield et al., 2015; Li et al., 2019). Thus, there appears to be two separate populations in PB_L_: one with an unknown identity that mediates caloric-sufficient satiety versus the CGRP^+^ population that mediates aversive appetite suppression.

In this study, we show that consumption of various highly palatable liquids and food activates a subset of neurons located in the caudal lateral subnucleus of the PB_L_ (PB_cl_) which are distinct from PB_L_^CGRP^ neurons. We used the activity-dependent capturing method called CANE (Sakurai et al., 2016; Rodriguez et al., 2017), to identify and characterize PB_cl_-palatable-food activated neurons (PANs). We further discovered that activation of these PB_cl_ PANs induces reward-like place preference behaviors yet decreases condensed milk consumption, while inhibition of these neurons increases milk consumption.

## Materials and Methods

### Animal statement

All experiments were conducted according to protocols approved by the Duke University Institutional Animal Care and Use Committee.

### Animals

Adult (P30–P60) male and female C57/BL6 mice (The Jackson Laboratory, stock 000664) were used for immunohistochemistry and *in situ* hybridization. Male and female Fos^TVA^ mice (Sakurai et al., 2016; The Jackson Laboratory, stock 027831) were used for capturing PB_cl_ PANs with the CANE technology, immunohistochemistry, and behavioral experiments for both ChR2 or TelC experimental and GFP control groups. All mice were housed in a vivarium with normal light/dark cycles in cages with one to five mice. A day before experiments, we singly housed mice. We used two exclusion criteria for our subjects: (1) poor recovery or other health concerns following surgical intervention or (2) missed injection or implantation target, as determined by histologic analysis. Animals were randomly selected from each litter. Random group allocation was maintained throughout the study, within constraints set by availability of in-house, purpose bred lines. Experimenter blinding was sufficient to control for selection bias. Furthermore, behavioral analysis relied on objective, automated measurements.

### Viruses

CANE-LV-Cre [titer, 5 × 10^8^ ifu/ml (Addgene plasmid, 86641); CANE-LV envelope (Addgene plasmid, 86666)] viruses were produced and packaged using HEK293T cells by co-transfecting a plasmid encoding the EnvA^M21^-VSVG (CANE-LV envelope) fusion envelope protein, the pLenti-hSyn-Cre-WPRE plasmid, and the psPAX2 plasmid into the cells. The CANE-LV envelope has three (R213A, R223A, R224A) mutations in the extracellular domain of the EnvA protein (Sakurai et al., 2016). AAV-CBA-Flex-ChR2(H134R)-mCherry (Atasoy et al., 2008; Addgene plasmid, 18916) and AAV-EF1α-flex-ChR2(H134R)-eYFP (Karl Deisseroth; Addgene plasmid, 20298) was purchased from the University of Pennsylvania Vector Core or from Addgene. AAV-CAG-flex-GFP was purchased from the University of North Carolina Gene Therapy Department. AAV-hSyn-Flex-TeLC-P2A-eGFP was produced and packaged using HEK293T cells by co-transfecting the AAV serotype plasmid AAV8, pAAV-hSyn-Flex,TeNT-P2A-GFP plasmid, and pAd.DELTA F6 plasmid into the cells (Zhang et al., 2015).

### Surgery

Animals were anesthetized with isoflurane in a stereotaxic frame (David Kopf Instruments) and small craniotomies were made over the target area. To target the caudal-lateral region of PB_L_, mice were mounted in the stereotaxic frame at an angle such that lambda was ∼180 μm ventral to bregma (in practice, 140–240 μm). The stereotaxic coordinates of virus injection and custom-made optic fiber (200-μm core diameter, Thorlabs) were AP –4.25 ± 0.15 mm, ML 1.35 ± 0.15 mm, and DV –3.1 ± 0.1 mm. The thin glass capillary was slowly lowered to the target site to minimize the brain injury. Virus was delivered into the target site at a flow rate of 100 nl/min using a pulled thin glass capillary (Warner Instruments) connected to an UltraMicroPump controlled by a SYS-Micro4 Controller 15 (World Precision Instruments).

**Figure 1.**
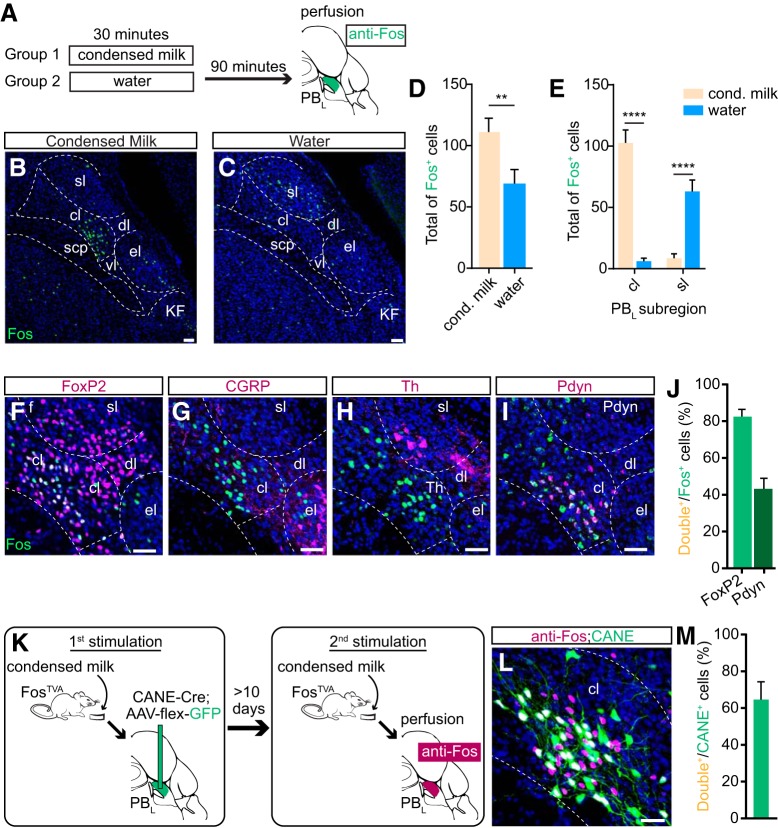
PB_cl_ neurons are activated by condensed milk consumption and are molecularly distinct from CGRP^+^ neurons. ***A***, Schematic illustration of Fos induction protocol. Ninety minutes after mouse consumed condensed milk or water *ad libitum* for 30 min, brainstem slices containing PB_L_ were stained for Fos expression. ***B***, ***C***, Representative images of Fos*^+^* neurons in PB_L_ after (***B***) condensed milk consumption and (***C***) water consumption. For representative images of Fos^+^ neurons in PB_L_ after consumption of other palatable liquid/food diets, see Extended Data Figure 1-1*A–E*. Small white dashed circles indicate subregions of PB_L_, including sl (superior lateral subnucleus), cl (caudal lateral subnucleus), dl (dorsal lateral subnucleus), el (external lateral subnucleus), vl (ventral lateral subnucleus), scp (superior cerebellar peduncles), KF (Koelliker–Fuse subnucleus). Blue, DAPI stain. Scale bars, 50 μm. ***D***, Total number of Fos^+^ neurons in PB_L_ of mice that consumed condensed milk (beige, *n* = 10 mice) and water (blue, *n* = 7 mice; two-tailed unpaired Student’s *t* test; **p = 0.007; *t*_(15)_ = 3.118). Data are mean ± SEM. ***E***, Numbers of Fos*^+^* neurons in PB_cl_ and PB_sl_ of mice that consumed condensed milk (beige, *n* = 10 mice) and water (blue, *n* = 7 mice; two-way ANOVA; PB_cl_: ****p < 0.0001; PB_sl_: ****p < 0.0001; *F*_(1,30)_ = 116.7). Data are mean ± SEM. ***F–I***, Staining of neurons activated by condensed milk consumption (anti-Fos, green) with (***F***) FoxP2*^+^* (immune), (***G***) CGRP*^+^* (immune), (***H***) Th*^+^* (immune), or (***I***) Pdyn^+^ (*in situ*) neurons in PB_cl_ (magenta) after condensed milk consumption. ***J***, Quantification of co-expression of Fos*^+^* neurons and FoxP2*^+^* and Pdyn^+^ neurons. Data are mean ±  SEM. ***K***, Schematic illustration of strategy to express GFP in PB_L_ neurons activated by condensed milk consumption (PANs) in Fos^TVA^ mice using CANE. For representative image and quantification of co-expression of Fos^+^ neurons and Pdyn^+^ neurons in PB_cl_ after chocolate Ensure consumption, see Extended Data Figure 1-1*F–H*. ***L***, The CANE method was used to capture PB_cl_ neurons activated by condensed milk consumption (green), and 10 d later, Fos was re-induced in PB_cl_ by a second bout of condensed milk consumption (magenta). Blue, DAPI. Scale bar, 20 μm. ***M***, The percentage of Fos*^+^* neurons among CANE^+^ neurons in PB_cl_. Data are mean ± SEM.

**Figure 2.**
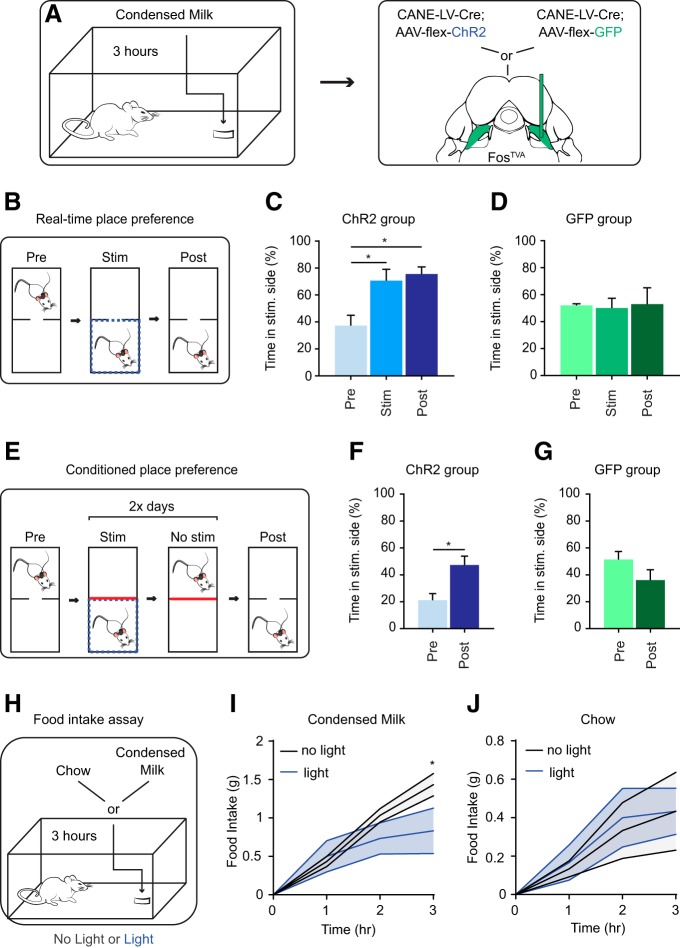
Optogenetic activation of PB_cl_ PANs induces place preference and decreases condensed milk consumption. ***A***, Schematic illustration of strategy to selectively express ChR2 or GFP in PB_cl_ PANs in Fos^TVA^ mice using CANE. ***B***, Schematic illustration of RTPP test. ***C***, Quantification of time the PAN-ChR2 group spent in preferred chamber before, during, and after optogenetic stimulation (*n* = 6 mice; one-way repeated measures ANOVA; *p = 0.01; *p = 0.04; *p* > 0.99; *F*_(1.46,7.29)_ = 9.22). Data are mean ± SEM. ***D***, Quantification of time the PAN-GFP group spent in preferred chamber before, during, and after optogenetic stimulation (*n* = 5 mice; one-way repeated measures ANOVA; *p* > 0.99; *p* > 0.99; *p* > 0.99; *F*_(1.42,5.69)_ = 0.05). Data are mean ± SEM. ***E***, Schematic illustration of CPP test. ***F***, Quantification of time the PAN-ChR2 group spent in preferred chamber before and after 2 d of optogenetic stimulation (*n* = 8 mice; two-tailed paired Student’s *t* test; *p = 0.02*; t*_(7)_ = 3.05) Data are mean ± SEM. ***G***, Quantification of time the PAN-GFP group spent in preferred chamber before and after 2 d of optogenetic stimulation (*n* = 5 mice; two-tailed paired Student’s *t* test; *p* = 0.19*; t*_(4)_ = 1.57) Data are mean ± SEM. ***H***, Schematic illustration of liquid/food intake assay. ***I***, Measured amount of condensed milk consumed at 1 h (*n* = 6 mice), 2 h (*n* = 3 mice), and 3 h (*n* = 3 mice) after condensed milk presentation with and without optogenetic stimulation (*p* > 0.99; *p* = 0.37; *p = 0.01; two-way repeated measures ANOVA; *F*_(3,14)_ = 4.38). Data are mean ± SEM. ***J***, Measured amount of regular chow consumed at 1 h (*n* = 6 mice), 2 h (*n* = 3 mice), and 3 h (*n* = 3 mice) after chow presentation with and without optogenetic stimulation (*p* > 0.99; *p* = 0.98; *p* = 0.92; two-way repeated measures ANOVA; *F*_(3,14)_ = 0.93). Data are mean ± SEM.

**Figure 3.**
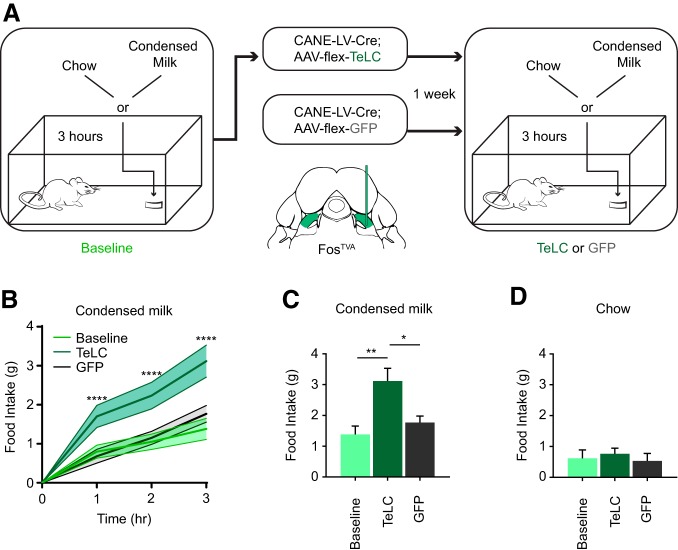
TeLC mediated silencing of PB_cl_ PANs increases condensed milk consumption. ***A***, Schematic illustration of the liquid/food intake assay before (baseline) and 7 d after selectively expressing TeLC or GFP in PB_cl_ PANs in Fos^TVA^ mice using CANE. ***B***, Measured amount of condensed milk consumed after 1, 2, and 3 h in PAN-TeLC group before (baseline) and after TeLC expression, and in a control PAN-GFP group (*n* = 6 mice per group; hr 1: ****p < 0.0001; hr 2: *****p < 0.0001; hr 3: *****p < 0.0001; two-way repeated measures ANOVA; *F*_(3,15)_ = 62.19). Data are mean ± SEM. ***C***, Total amount of condensed milk consumed after 3 h (*n* = 6 mice per group; baseline compared to TeLC: **p = 0.004; baseline compared to GFP: *p* > 0.99; TeLC compared to GFP: *p = 0.02; one-way ANOVA; *F*_(2,15)_ = 8.61). Data are mean ± SEM. ***D***, Amount of regular chow consumed 3 h after chow presentation (*n* = 6 mice per group; baseline compared to TeLC: *p* > 0.99; baseline to GFP: *p* > 0.99; TeLC to GFP: *p* > 0.99; one-way ANOVA; *F*_(2,15)_ = 0.25). Data are mean ± SEM.

The injected viruses and the waiting period for viral transgene expression for the different experiments are. For experiments in Figure 1, CANE-LV-Cre (500 nl) together with AAV-CAG-flex-GFP (300 nl), waiting >10 d. For experiments in Figure 2, CANE-LV-Cre (500 nl) together with AAV-CAG-flex-GFP (300 nl), AAV-CBA-Flex-ChR2(H134R)-mCherry or AAV-EF1α-DIO-hChR2-eYFP (300 nl), waiting six to eight weeks. For experiments in Figure 3, CANE-LV-Cre (500 nl) together with AAV-CAG-flex-GFP (300 nl) or AAV-hSyn-Flex-TeLC-P2A-eGFP (300 nl), waiting 7 d.

10.1523/ENEURO.0252-19.2019.f1-1 Extended Data Figure 1-1 PBcls are activated by high-caloric palatable liquids and food. ***A***, Schematic illustration of Fos induction protocol. Ninety minutes after mouse consumed condensed chocolate Ensure/peanut butter/sucrose/sucralose *ad libitum* for 30 min, brainstem slices containing PB_L_ were stained for Fos expression. ***B***, ***C***, Representative images of Fos*^+^* in PB_L_ after (***B***) chocolate Ensure consumption (***C***) peanut butter consumption (***D***) sucrose consumption (***E***) sucralose consumption. Small white dashed circles indicate subregions of PB_L_, including sl (superior lateral subnucleus), cl (caudal lateral subnucleus), dl (dorsal lateral subnucleus), el (external lateral subnucleus), vl (ventral lateral subnucleus), scp (superior cerebellar peduncles), and KF (Koelliker–Fuse subnucleus). Blue, DAPI stain. Scale bars, 50 μm. ***F***, Staining of neurons activated by chocolate Ensure consumption (anti-Fos, green) with *PDYN^+^* (*in situ*) neurons in PB_cl_ (magenta) after chocolate Ensure consumption. ***G***, Quantification of co-expression of Fos*^+^* neurons and *PDYN^+^* neurons. Data are mean ± SEM. Download Figure 1-1, EPS file.

### Immunohistochemistry

All mice were deeply anesthetized with isoflurane, and then transcardially perfused with ice-cold 4% paraformaldehyde in 0.1 M phosphate buffer, pH 7.4 (4% PFA). Dissected brain samples were then postfixed overnight in 4% PFA at 4°C, cryoprotected in a 20% sucrose solution in PBS at 4°C, frozen in Tissue-Tek O.C.T. compound (Sakura, 25608-930) and stored at –80°C until sectioning. All coronal brain sections were sliced at 60–80 μm. The serial brain sections were collected in a 24-well plate and washed with PBS three times. The sections were blocked with 2% bovine serum albumin (BSA) in PBS with 0.3% Triton X-100 (blocking solution) at room temperature for 1 h. The sections were treated with primary antibody in blocking solution at 4°C. for overnight. The sections were washed three times followed by secondary antibody treatment at 4°C for 2 h. Sections were counter-stained with NeuroTrace fluorescent Nissl stain (fluorescent Nissl stain; Invitrogen, N-21479) or 4’,6-diamidino-2-phenylindole (DAPI; Sigma, D9564). After this incubation, sections were washed and mounted. The primary antibodies used in this study are: goat anti-Fos (Santa Cruz Biotechnology, sc52-g, 1:300), rabbit anti-CGRP (Röhn, 2011; Millipore, AB15360, 1:1000), sheep anti-FoxP2 (Geerling et al., 2011; R&D Systems, AF5647, 1:5000), rabbit anti-Th (Millipore, MAB318, 1:1000). The secondary antibodies are: Alexa Fluor 488 donkey anti-goat (Jackson ImmunoResearch, 705-545-147, 1:1000), Cy3 donkey anti-goat (Jackson ImmunoResearch, 705-165-147, 1:1000), and Cy3 donkey anti-rabbit (Jackson ImmunoResearch, 711-165-152, 1:1000).

### Floating section *in situ* hybridization

Floating section *in situ* hybridization was performed following a previously described protocol (Stanek et al., 2016; Bellavance et al., 2017). All mice were deeply anesthetized with isoflurane, and then transcardially perfused with ice-cold 4% PFA in 0.1 M phosphate buffer, pH 7.4. Dissected brain samples were then postfixed overnight in 4% PFA at 4°C, cryoprotected in a 20% sucrose solution in PBS in DEPC (Sigma-Aldrich, D5758) treated double-deionized water at 4°C, frozen in Tissue-Tek O.C.T. Compound and stored at –80°C until sectioning. For each mouse, 60-μm sections containing the PB_L_ were collected with a cryostat at –20°C and resuspended in 4% PFA. Samples were rinsed with three times with PBS in DEPC water (DEPC PBS), washed with 0.3% Triton X-100 in DEPC PBS, treated with 5 μg/ml protinease K in DEPC PBS, acetylated using a solution of 0.465-g TEA, 28-μl 10 N NaOH, 25-ml DEPC water, and 62.6-μl acetic anhydride. Samples were incubated for 1 h at 60°C in a prehybridization buffer solution containing 0.2× SSC, 10% blocking reagent (Sigma-Aldrich), 50% formamide, 2% N-lauroylsarcosine (NLS), 10% SDS for 60 min. This was followed by hybridization of fluorescein isothiocyanate-labeled *Fos* and digoxigenin-labeled prodynorphin probes which were prepared by amplifying cDNA fragments of *Fos* and prodynorphin by PCR with the antisense primers containing the T7 promoter sequence. *In vitro* transcription was then performed from the PCR-amplified template using T7 RNA polymerase (Roche, 10881767001) for the synthesis of the antisense probes (Sakurai et al., 2016). Samples were incubated with probes overnight in hybridization buffer at 60°C. The following day, samples were washed in prehybridization buffer at room temperature. Samples were incubated at room temperature in a 10% blocking solution in TBST, followed by application of 1st primary antibody (anti-DIG-AP 1:3500, Roche, 11093274910) overnight at 4°C. The following day, samples were washed with TBST, followed by 100 mm Tris-HCl, pH 8.0 and hybridization signals for prodynorphin were visualized using FastRed (Sigma-Aldrich, F4648) for 90 min. This was followed by application of 2nd primary antibody (POD anti-FITC 1:500, Roche 1426346910) at RT for 1 h, and hybridization signals for *Fos* were visualized using FITC-TSA (Sigma-Aldrich, F4648) for 10 min. After one wash in PBS-DAPI (1:5000) for 5 min and two washes in buffer solution, sections were mounted on glass slides.

### Image acquisition and quantification

Samples were imaged using a Zeiss 700 laser scanning confocal microscope. *In situ* samples were imaged at 20× resolution at three *z*-positions. All *z*-positions for each slice were merged into a single image in Adobe Photoshop CS6 for quantification. All other samples were imaged at 10× resolution. The captured neurons and Fos expressing neurons in all immunohistochemistry and *in situ* hybridization experiments were manually counted, and percentages were calculated within each animal before averaging percentages across animals.

### Behavioral experiments for Fos immunostaining

Adult male and female C57/BL6 mice at ages more than six weeks were singly housed at least 1 d and water restricted overnight before receiving either an appetitive or neutral liquid, or food restricted overnight before receiving peanut butter. For visualizing Fos expression induced by highly-palatable liquid/food consumption, mice were given diluted condensed milk (Nestle Carnation), milk chocolate nutrition shake (Ensure Plus), peanut butter (Jif To Go creamy natural peanut butter), 146 mM sucrose (Calbiochem, 8510), 1 mM sucralose (Sigma-Aldrich, 90984), or water in their home cage for 30 min. Ninety minutes later, the animals were perfused as described in the method for immunostaining above. The nutritional composition of 1 ml (two calories) of 50:50 condensed milk is: 0.09-g total fat (0.04-g saturated fat; 21% of calories), 0.65mg sodium, 0.37 g total carbohydrates (0.37-g sugars; 67% of calories) 0.05-g total protein (12% of calories). The nutritional composition of 1 ml (one calorie) of chocolate ensure is: 0.04-g total fat (0.01-g saturated fat, 0.02-g polyunsaturated fat, 0.02 monounsaturated fat, 0.04-mg cholesterol; 28% of calories), 0.9-mg sodium, 2.41-mg potassium, 0.22-g total carbohydrates (<0.01-g dietary fiber, 0.09-g sugars; 57% of calories), 0.05-g protein (15% of calories). The nutritional composition of 1 g (5.81 calories) of peanut butter is: 0.47-g total fat (0.1-g saturated fat; 15% of calories), 2.44-mg sodium, 0.26-g total carbohydrates (0.07-g dietary fiber, 0.12-g sugars; 17% of calories), 0.23-g protein (68% of calories). The nutritional composition of 1 ml (0.2 calories) of 146 mM (5%) sucrose in water is: 0.05-g total carbohydrates (0.05-g sugars; 100% of calories). The nutritional composition of 1 ml (0.1 calories) of 1 mM (0.04%) sucralose in water is: 0.04-g total carbohydrates (0.03-g sugars; 100% of calories).

### Behavioral experiments for capturing PB_cl_ PANs with CANE virus

The CANE capturing method is as follows (Sakurai et al., 2016). In Fos^TVA^ mice, activated neurons transiently express *Fos* which induces expression of a destabilized TVA (dsTVA) receptor. Lentivirus pseudotyped with an engineered mutated envelope protein (CANE envelope) specifically binds cells expressing high-level TVA receptor, which are strongly Fos^+^ neurons. In this way, CANE viruses selectively infect Fos^+^ neurons and deliver desired transgenes to be expressed in Fos^+^ neurons.

Here, adult male and female Fos^TVA^ mice at ages more than six weeks were singly housed for at least 1 d and water restricted overnight and were given diluted condensed milk in their home cage for 30 min; 60–90 min later, mice were anesthetized and underwent stereotaxic surgery for CANE-virus injection.

### Optogenetic activation of CANE-captured PB_cl_ PANs in a real-time place preference (RTPP) test and liquid/food intake assay

Channelrhodopsin (ChR2^+^) or control (GFP^+^) protein was expressed in CANE-Cre^+^ PB_cl_ PANs by injection of either AAV-CBA-Flex-ChR2(H134R)-mCherry, AAV-EF1α-DIO-hChR2-eYFP or AAV-CAG-Flex-GFP in adult Fos^TVA^ mice (as described above). Three weeks later, virus injected mice were implanted with custom made optic fibers which were placed above PB_cl_ on both sides and fixed on the skull with dental cement (Parkell). One week later, the animals were subjected to a two-chamber RTPP test in light cycle, using a modified method described in previous studies (Stamatakis and Stuber, 2012; Jennings et al., 2013). The custom-made behavior chamber is 50.1 × 27.7 × 31.2 cm (W × L × H), made with clear acrylic Plexiglas that had distinct stripe patterns in each chamber. For optogenetic stimulation, laser is delivered through patch cables attached to the implanted optic fiber. The RTPP test is as follows. The mouse is placed in the center of the box and allowed to explore both chambers without light stimulation (prestimulation) for 10 min. Generally, after exploration, the mouse shows a small preference for one of the two chambers. Subsequently, blue light stimulation (20 Hz, 20-ms pulse width, ∼3.5 mW) is delivered whenever the mouse enters or stays in the non-preferred chamber, and light is turned off when the mouse moves to the other chamber (stimulation phase, total 10 min). Finally, the mouse can freely explore both chambers without blue light stimulation (poststimulation) for 10 min. We recorded behavioral data via a webcam (Logitech web-camera, PN 960-000764) interfaced with Bonsai software (Lopes et al., 2015). Real-time laser stimulation was controlled by Bonsai software through Arduino with a custom-made Arduino sketch (Arduino UNO, A00073).

After one week, the same group of mice were subjected to another behavioral test, where animals were subjected to a two-chamber classic conditioned place preference (CPP) test in same behavior chamber used for RTPP. The mouse is first habituated to the chamber on day 1. On day 2, the mouse is placed in the center of the box and allowed to explore both chambers without light stimulation (prestimulation) for 10 min. Generally, after exploration, the mouse shows a small preference for one of the two chambers. In the following 2 d (day 3 and day 4), the mouse is closed off in the preferred chamber with no stimulation for 30 min in the morning, and then closed off in the non-preferred chamber with blue light stimulation (20 Hz, 20-ms pulse width, ∼3.5 mW) for 30 min in the afternoon. On the final day (day 5), the mouse can explore both chambers without blue light stimulation (poststimulation) for 10 min, and their behaviors are recorded and analyzed.

After one week, the ChR2^+^ group underwent a liquid/food intake assay in their home cage within 6–9 P.M. (normal feeding cycle), using a modified method as previously described (Garfield et al., 2015; Fenselau et al., 2017; Li et al., 2019). The liquid/food intake assay is as follows: Mice were given a measured amount of diluted condensed milk or regular mouse chow for 3 h with optogenetic blue light stimulation (5 s on and 1 s off, 20-ms pulses, 20 Hz, ∼3.5 mW). The order of the liquid or food was randomized among the groups. The amount of liquid or food ingested was measured as the difference between the final measurement and the initial measurement. After completion of the photostimulation experiments, mice were perfused, and the locations of optic fiber tips were identified.

### Genetic silencing of CANE-captured PB_cl_ PANs in a liquid/food intake assay

Tetanus toxin (TeLC) or control GFP was expressed in CANE-Cre^+^ PB_L_-satiety neurons by injection of either AAV-hSyn-Flex-TeLC-P2A-eGFP or AAV-CAG-Flex-GFP in adult Fos^TVA^ mice (as described above). Three days before virus injection (baseline), the experimental group was subjected to the liquid/food intake assay in their home cage from 6–9 P.M. (normal feeding cycle), where they were given a measured amount of diluted condensed milk or regular mouse chow. The order of the liquid or food was randomized among the groups. The amount of diluted condensed milk or water was measured every hour for 3 h. This assay was repeated for both TelC and control groups 8 d after virus injection (TeLC, control). The amount of liquid or food ingested was measured as the difference between the measurements taken at each hour and the initial measurement. After completion of the silencing experiments, mice were perfused, and viral expression was assessed.

### Statistical analysis

All data are presented as mean ± SEM. Statistical analysis of data were conducted and graphed using GraphPad Prism 8 (Prism software). Significance was set at *p* < 0.05. All data were normally distributed as determined by a Shapiro–Wilk normality test. For Figure 1*D*, unpaired Student’s *t* test was used to compare milk and water groups. For Figure 1*E*, two-way ANOVA of treatment over brain region was used with a Bonferroni *post hoc* test to assess differences in Fos expression. For Figure 2*F*,*G*, paired Student’s *t* tests were used to compare before and after optogenetic stimulation. For Figure 2*C*,*D*, one-way repeated measures ANOVA of before, during, and after optogenetic-stimulation was used followed by a Bonferroni *post hoc* test to assess changes in time spent in preferred side. For Figures 2*I*,*J*, 3*B* , two-way repeated measures ANOVA of mouse group and time was used followed by a Bonferroni *post hoc* test to assess changes in liquid or food intake over time. For Figure 3*C*,*D*, one-way ANOVA of mouse group over liquid or food intake was used followed by a Bonferroni *post hoc* test. Relevant values used for statistical analysis are included in the figure legends as follows: (*t* test) *t* subscript degrees of freedom = *t* statistic; (ANOVA) *F* subscript between-groups degrees of freedom, within groups degrees of freedom = *F* statistic.

### Data availability

The data collected in this study are available from the corresponding author on request.

## Results

### Consumption of highly palatable foods activates PB_cl_ neurons

Mice were given access to drink from either a bottle containing water, a non-caloric liquid, or a bottle containing 50% condensed milk, a high-caloric palatable liquid food, alone, for 30 min after overnight water restriction, euthanized, and brain sections were immunostained for expression of immediately-early gene *Fos* as a marker for activated neurons in PB_L_ (Fig. 1*A*). Drinking condensed milk activated neurons in PB_L_, resulting in significantly more Fos^+^ neurons than drinking water (condensed milk, 116.4 ± 9.83, and water, 69 ± 11.52 total Fos^+^ neurons; *p* = 0.007; *n* = 10, 7; Fig. 1*B*,*C*). Furthermore, drinking condensed milk induced robust Fos^+^ expression specifically in PB_cl_, whereas drinking water preferentially activated neurons in the superior lateral subnucleus of the PB_L_ (PB_sl_; PB_cl_: condensed milk 105.3 ± 8.93, water 6 ± 2.61 Fos^+^ neurons; *p* < 0.0001; *n* = 10, 7; and PB_sl_: condensed milk 11.1 ± 3.37, water 63 ± 9.28 Fos^+^ neurons; *p* < 0.0001; *n* = 10, 7; Fig. 1*B–D*). To determine whether PB_cl_ neurons were activated by intake of other palatable liquid or food, separate groups of mice were given access to either chocolate Ensure, peanut butter, 146 mM sucrose dissolved in water, or 1 mM sucralose dissolved in water (Extended Data Fig. 1-1*A*). Drinking chocolate Ensure, another high-caloric palatable liquid, also induced robust Fos^+^ expression specifically in PB_cl_ (Extended Data Fig. 1-1*B*). Interestingly, consuming peanut butter, a high-caloric solid food, induced robust Fos^+^ expression in the same PB_cl_ region (Extended Data Fig. 1-1*C*). In contrast, drinking either sucrose or sucralose, which are low- or non-caloric sweet tastants, preferentially activated neurons in PB_sl_, but not in PB_cl_ (Extended Data Fig. 1-1*D*,*E*). These results are similar to that observed for drinking water. In other words, non-caloric and caloric liquid intakes are regulated by distinct circuits in PB_L_, a result consistent with a recent finding Oxtr^+^ PB_L_ neurons selectively regulating non-caloric fluid drinking (Ryan et al., 2017). Therefore, we called these PB_cl_ neurons palatable-food activated neurons (PANs). Notably, a previous study showed that the satiety-signaling PVH^MC4R^ afferents terminate in the PB_cl_ region where the PANs are located (Shah et al., 2014; Garfield et al., 2015; Li et al., 2019).

### PB_cl_ PANs are distinct from CGRP^+^ PB_el_ neurons

Forkhead box protein P2 (FoxP2) transcription factor was previously shown to be primarily expressed in dorsal subregions of PB_L_ (Geerling et al., 2011; Gaub et al., 2016). Using two-color immunofluorescence, we found that a majority of Fos^+^ PB_cl_ PANs expressed FoxP2, but they represented a small fraction of the total FoxP2^+^ PB_L_ cells (83.19 ± 3.29 of total Fos^+^ PB_cl_ PANs were FoxP2*^+^*; *n* = 4; Fig. 1*F*,*J*). We next determined whether Fos^+^ PB_cl_ PANs express CGRP and found that there was no overlap (0 of total Fos^+^ PB_cl_ PANs were CGRP*^+^*; *n* = 4; Fig. 1*G*). This finding suggests that these Fos^+^ PB_cl_ PANs are likely functionally distinct from anorexigenic PB_L_^CGRP^ neurons (located in the external lateral part of PB_L_).

We next consulted the Allen Brain Atlas to find other potential genetic markers enriched in PB_cl_ subregion. Two candidate genes were identified: tyrosine hydroxylase (*Th*), which encodes the enzyme regulating the synthesis of the neurotransmitter dopamine, and prodynorphin (*Pdyn*), which encodes the neuropeptide dynorphin and is previously shown to be expressed in cells implicated in thermo-sensation (Geerling et al., 2011). Using immunohistochemistry, we found that *Fos* ^+^ PB_cl_ PANs do not express Th (0 of total *Fos* ^+^ PB_cl_ PANs were Th*^+^*; *n* = 4; Fig. 1*H*,*J*). Interestingly, using two-color *in situ* hybridization, we found that ∼44% of *Fos* ^+^ PANs activated by condensed milk consumption and 38% of *Fos* ^+^ PANs activated by chocolate Ensure consumption were *Pdyn^+^* [43.62 ± 8.16; *n* = 3 (Fig. 1*I*,*J*); 38 ± 5.55; n = 4 (Extended Data Fig. 1-1*F–H*)], suggesting the possibility that dynorphin is released on consumption of highly caloric palatable liquids.

### CANE is efficient and selective in activity-dependent capture of PB_cl_ PANs

To determine the functional role of Fos^+^ PB_cl_ PANs in liquid and food consummatory behaviors, we needed to label and manipulate these cells specifically. Since we did not identify any molecular markers that are expressed by all PANs and are only expressed in PANs, we therefore used an activity-dependent method called CANE, to virally tag transiently activated neurons in the genetically engineered Fos^TVA^ mice (Sakurai et al., 2016; Rodriguez et al., 2017). We first determined whether CANE could indeed selectively and efficiently label PB_cl_ PANs. In a two-bout experimental paradigm, CANE was used to capture PB_cl_ neurons activated by drinking condensed milk through co-injection of CANE-LV-Cre and AAV-flex-GFP into the PB_cl_. Three weeks later, the same animal drank milk again to induce Fos^+^ expression and was then anesthetized and killed to obtain samples for immunostaining (Fig. 1*K*). In the milk–milk condition, 64.66 ± 4.3% (*n* = 5) of CANE-captured cells were Fos^+^ (Fig. 1*L*,*M*). This indicated that the second condensed milk consumption reactivated many (∼65%) of the same cells excited by the first consumption. Thus, CANE is sufficiently specific and efficient to tag PANs.

### Optogenetic activation of PB_cl_ PANs neurons elicits place preference

Given that PB_cl_ PANs are activated by consuming high-caloric palatable liquids and solid food, we asked whether artificially activating these cells will produce a positive feeling. To test this, we used CANE to express either channelrhodopsin or GFP (control) in PB_cl_ PANs, and subjected mice to a modified RTPP assay, which has been used in recent studies to assay affective behavior (Stamatakis and Stuber, 2012; Jennings et al., 2013). Optic fibers were implanted bilaterally above PB_cl_ in either PAN-ChR2 mice (*n* = 6) or PAN-GFP mice (*n* = 5; Fig. 2*A*). Mice were habituated and placed in a two-chamber arena. Their behaviors were recorded under three conditions: (1) freely exploring with no stimulation for 10 min (baseline), followed by (2) 10 min of conditioned photoactivation when the mouse is in its non-preferred chamber (stimulation), and followed again by (3) 10 min without stimulation (poststimulation; Fig. 2*B*). On photo-stimulation PAN-ChR2 mice spent significantly more time on the stimulated side (pre: 37.31 ± 7.61%, stim: 70.63 ± 8.38%, *p* = 0.01, *n* = 6; Fig. 2*C*). In the poststimulation period, all stimulated mice still showed preference of the chamber in which they received photostimulation (pre: 37.31 ± 7.61%, post: 75.58 ± 5.24%, *p* = 0.04, *n* = 6; Fig. 2*C*). Light illumination had no effect on movement and behavior of the PAN-GFP mice (pre: 52.08 ± 1.23%; stim: 50.11 ± 7.19%, post: 52.92 ± 12.17%, *p* > 0.99, *n* = 5; Fig. 2*D*). These results suggest that the optogenetic stimulation of the PB_cl_ PANs caused an appetitive effect, indicating that the neurons likely encode a positive emotional valence.

We further wanted to determine whether optogenetic activation would be sufficient to induce a longer lasting positive memory using the conventional CPP assay. Mice were habituated first by placing them in the two-chamber arena and allowing free exploration. Subsequently, they were subjected to 2 d of conditioning: mice were paired with photostimulation in the non-preferred chamber for 30 min, and 8 h later, they were placed in the preferred chamber without light stimulation for 30 min. On the fourth day, they explored the arena freely in the absence of light stimulation for 10 min (poststimulation; Fig. 2*E*). PAN-ChR2 mice (*n* = 8) spent significantly more time in the chamber where they were photostimulated previously (pre: 21.12 ± 4.912, post: 47.27 ± 6.541, *P* = 0.02; Fig. 2*F*). Light illumination had no effect on the movement and behavior of the control PAN-GFP mice (*n* = 5; pre: 51.46 ± 5.86, post: 36.01 ± 7.753, *p* = 0.19; Fig. 2*G*). These results suggest that optogenetic activation of PB_cl_ PANs produced a preference memory.

### Optogenetic Activation of PB_cl_ PANs neurons decreases condensed milk consumption

Previous studies have shown that activation of PVH^MC4R^ afferents in PB_cl_ region induced homeostatic regulated satiety (Garfield et al., 2015; Li et al., 2019). Therefore, we wanted to determine whether activating CANE-captured PANs would also affect liquid and food intake. PAN-ChR2 mice (*n* = 6) underwent a caloric liquid/food intake assay in their home cage during their normal feeding cycle, where they were given a measured amount of condensed milk or regular mouse chow, which is less palatable, for 3 h either without (baseline) or with optogenetic stimulation (Fig. 2*H*; Garfield et al., 2015; Fenselau et al., 2017; Li et al., 2019). At 1 h after the start of feeding, the amount of condensed milk ingested was not different between the two conditions (with or without light stimulation; *n* = 6; no light, 0.43 ± 0.07; light, 0.5 ± 0.2; *p* > 0.99; Fig. 2*I*). However, when measured after 3 h, the total amount of condensed milk ingested in the PAN-activated condition was significantly less (*n* = 3; no light, 1.43 ± 0.15; light, 0.83 ± 0.3; *p* = 0.01; Fig. 2*I*). Light illumination had no effect on the consumption of regular chow either measured after 1 h (*n* = 6; no light, 0.13 ± 0.04; light, 0.17 ± 0.09; *p* > 0.99; Fig. 2*J*) or after 3 h (*n* = 3; no light, 0.43 ± 0.2; light, 0.43 ± 0.12; *p* > 0.99; Fig. 2*J*). These results suggest that activation of PB_cl_ PANs is sufficient to specifically reduce the consumption of highly caloric and appetitive condensed milk, but not the regular chow, over a long duration. These results complement the previous findings revealing the homeostatic role of activating PVH^MC4R^-PB_cl_ pathway in reduction of liquid/food intake (Shah et al., 2014; Garfield et al., 2015; Li et al., 2019).

### TeLC-mediated silencing of PB_cl_ PANs increases condensed milk consumption

We next asked whether silencing the PANs would increase caloric liquid or food consumption. We used CANE to express either TeLC or GFP in PB_cl_ PANs. TeLC abolishes synaptic transmission from PANs. PAN-TeLC (*n* = 6) and PAN-GFP mice (*n* = 6) underwent the liquid/food intake assay in their home cage during their normal feeding cycle before (baseline) and after CANE-mediated expression of TeLC or GFP (Fig. 3*A*). We observed a steady increase of condensed milk intake occurring 5–7 d after TeLC expression (data not shown). This increase in condensed milk intake plateaued after 7 d. On day 7, we observed a significant increase in condensed milk intake compared to Baseline, and compared to PAN-GFP mice at 7 d after GFP expression (hour 1–3: *p* < 0.0001; total milk intake: baseline vs TeLC; *p* = 004; GFP vs TeLC; *p* = 0.02; Fig. 3*B*,*C*). Although we saw momentary body weight gain during the 3-h access to condensed milk, we did not observe an overall increase in body weight 7 d after TeLC expression (data not shown), and we did not test the animals over long-term on a high-caloric diet. There was no difference in condensed milk intake between PAN-GFP mice and baseline of PAN-TeLC mice before TeLC was expressed (total milk intake: baseline vs GFP; *p* > 0.99; Fig. 3*B*,*C*). Interestingly, there was no significant difference across all groups in regular chow intake (baseline vs TeLC, *p* > 0.99; control vs TeLC, *p* > 0.99; baseline vs control, *p* > 0.99; Fig. 3*D*). These results suggested that PB_cl_ PANs is specifically required for preventing over consumption of a high-caloric palatable diet, while other pathways (see Discussion) are sufficient for signaling satiety from regular food.

## Discussion

Here, we molecularly and functionally characterized an ensemble of neurons located within PB_cl_ that likely relay appetitive and satiating signals after consuming highly caloric liquid and food diets. In previous studies, it was discovered that PVH^MC4R^ neurons regulate satiety and prevent over-feeding and consequent obesity (Balthasar et al., 2005). PVH^MC4R^ axons innervate PB_cl_ (Shah et al., 2014; Garfield et al., 2015). Optogenetic activation of PVH^MC4R^ -PB_cl_ axons reduced food intake and induced positive affective behavior (Garfield et al., 2015). These and other studies have implied that neurons in PB_cl_ play a role in homeostatic caloric-dependent satiety in sated states, but the identities of the caloric-dependent satiety-signaling neurons in PB_cl_ were unknown (Balthasar et al., 2005; Shah et al., 2014; Garfield et al., 2015; Li et al., 2019). Using a combination of activity-dependent tagging, *in vivo* optogenetic activation, and TeLC silencing experiments, we uncovered palatable food activated neurons in PB_cl_ that are molecularly and functionally distinct from CGRP^+^ anorexigenic neurons located in PB_el_ and spatially and functionally distinct from Oxtr^+^ non-caloric fluid regulating neurons located in PB_sl/dl_ (Campos et al., 2016; Ryan et al., 2017).

A total of 44% of PB_cl_ PANs expressed *Pdyn* suggesting that a heterogeneous population of PANs work together to regulate satiety of highly caloric food in sated states. Optogenetic activation and TeLC silencing of PANs bidirectionally regulated consumption of condensed milk, a highly caloric liquid diet, but not regular chow. Therefore, it is possible that PANs are a subpopulation of a caloric-dependent satiety regulating ensemble in PB_cl_. Further molecular characterization, potentially using RNA sequencing in combination with the CANE strategy, may be required to reveal the full ensemble of caloric-dependent satiety-signaling neurons in PB_cl_. This result also demonstrates the selectivity of the CANE method in capturing PB_cl_ neurons activated by consumption of condensed milk. Nevertheless, it has not been determined whether these CANE-captured PB_cl_ PANs neurons are the same PB_cl_ population implicated in receiving satiety-related interoceptive information directly from PVH^MC4R^ neurons. Based on the anatomic location of PANs and their functional role in caloric-dependent satiety, activity-dependent transsynaptic tracing may reveal that majority of these neurons do receive direct input from caloric-dependent satiety regulating PVH^MC4R^ neurons. Thus, future activity-dependent capturing studies are necessary for a detailed analysis and characterization of the PVH^MC4R^-PB_cl_ satiety-regulating pathway.

Our finding that consumption of high caloric liquid and foods preferentially activates PB_cl_ neurons that signal satiety maintenance sheds some light on a complex circuit of PB_L_ neurons signaling different facets of liquid and food intake regulation: caloric versus non-caloric, liquid versus food, nutritional composition, sated versus hunger states, and appetitive versus aversive. Recently, multiple parallel PB_L_ circuits have been discovered to be critical regulators of satiety. Although the PVH^MC4R^ -PB_cl_ circuit plays a role in regulating satiety in a sated state, these neurons comprise of ∼50% of PVH neurons that mediate satiety. Recently, it was shown that the other ∼50% of PVH neurons, which expressed *Pdyn* and project to the prelocus coeruleus (preLC), similarly regulate satiety in sated states (Li et al., 2019). In parallel, a group of Oxtr^+^ neurons located in PB_sl/dl_ specifically regulate non-caloric fluid intake, but not caloric-dependent satiety (Ryan et al., 2017). Together, some of these distinct PB_L_ and preLC circuits (i.e., Pdyn^+^ caloric-signaling neurons and Oxtr^+^ neurons fluid intake-signaling neurons) may act together for integrated food and fluid intake regulation. Other PB_L_ circuits, however, may act antagonistically with one another in regards to the valence of food intake (i.e., Pdyn^+^ appetitive-signaling neurons and CGRP^+^ malaise-signaling neurons). Further work investigating how much these circuits contribute in working synergistically or antagonistically is critical to understanding PB_L_’s role within the complex fluid/food regulating circuit.

The PB_cl_ PANs were identified through Fos expression after condensed milk consumption. Although Fos is a surrogate for elevated neural activity, it lacks temporal resolution and does not give any information about second-to-second neural dynamics. Therefore, it is unclear when PB_cl_ neural activity increases during consumption. Future studies can take advantage of the CANE method to express a genetically encoded calcium indicator (such as GCaMP), and use *in vivo* calcium imaging (Ghosh et al., 2011; Chen et al., 2013) to track the activity of these cells in real-time while the mouse is consuming various liquids or foods (e.g., water, sucrose, condensed milk, chow) in a multitude of contexts and states (e.g., hungry vs sated). These techniques will shed light on the population dynamics of PB_cl_ cells in appetite/satiety regulation, whether and when the magnitude or firing rate of neural response to appetitive liquid and food consumption changes, and if there are subsets of neurons firing at distinct time points throughout feeding. Overall, the findings in this study further advanced our understanding of the neural circuit regulating satiety mediated termination of feeding. Future studies should take advantage of these PB_cl_ PANs to identify their downstream targets and elucidate how signals from PANs are used to control feeding behaviors.

## Synthesis

Reviewing Editor: Alexxai Kravitz, Washington University in St. Louis Decisions are customarily a result of the Reviewing Editor and the peer reviewers coming together and discussing their recommendations until a consensus is reached. When revisions are invited, a fact-based synthesis statement explaining their decision and outlining what is needed to prepare a revision will be listed below. The following reviewer(s) agreed to reveal their identity: Matthew Carter, Thomas Kash.

Your manuscript has been read by myself and two reviewers. We all agreed that this is a well done study with appropriate controls that is a strong candidate for publication in eNeuro. However, there were a few issues that should be addressed before publication:

1) Reviewer #1 noted several methodological details that were insufficient and should be expanded on. The following should be addressed:

a) What was the nutritional composition and source of the condensed milk? No information is provided in the Methods as to carbohydrate/fat/protein content.

b) Were the animals drinking the condensed milk? It was hard to tell if the condensed milk was in liquid or solid form from reading the manuscript. If a liquid, then why didn't consumption of condensed milk also induce Fos expression in the PBsl, as water does?

c) In the Methods, the authors often omit procedures and instead refer readers to previous papers (e.g. virus production procedures on p.5 or design of in situ hybridization probes on p. 7. Please do not make readers go on a hunt for procedures salient to the present study and instead re-write these methods in the current Methods section.

2) Several comments were made to increase clarify and accuracy of their descriptions:

a) It seems an overreach to suggest that PBcl neurons regulate a “pleasurable” satiety because only one stimulus was presented, and it happens to be a high caloric and appetitive stimulus-condensed milk.

b) The term “Milk Activated Neurons (MANs)” seems oddly specific, as these neurons are likely activated by multiple stimuli (not just milk). If standard chow does not activate these neurons, can they really be considered to induce a “pleasurable” satiety? Or is it more that the food is appetitive and the animals are responding to the flavor or the caloric content? This manuscript would make a more specific conclusion if instead of Milk Activated Neurons, they could describe specifically what these neurons respond to by examining Fos expression following different nutritional molecules (e.g. sugars, fats) or caloric parameters (e.g. refeeding after a fast).

c) The title of the manuscript refers to a parabrachial “microcircuit.” It seems inappropriate to refer to the focus on PBcl neurons as a “microcircuit,” as “circuit” implies a projection between two populations of neurons and “micro” implies a circuit within a local area. Because this paper only focuses on the PBcl, the term “microcircuit” is not justified.

3) The authors use an irreversible TeLC method to silence PBcl neurons. This is a great strategy, but it is curious that the authors only did a very short term experiment on the order of hours. It would be very interesting to know the behavior of these mice over the long-term, as PBcl silencing is long-term. Does an increase in food intake occur over several days/weeks? Does body weight continuously increase? The authors don't seem to be fully utilizing the amount of work they have put into generating these mice. More information on this should be included if possible, or a statement on why it is not possible.

Additional comments were made, but the reviewers and I decided these were not strictly necessary for publication, so I include them here for your information, and to encourage you to include this data if you already have it or it can be easily collected.

1) A stronger attempt could be made to understand the identify of the MANs. The authors consistently refer to the fact that PVN-MC4R neurons project to the PBcl and that the neurons under present investigation are likely the same target neurons studied by Garfield et al. (2015) and Shah et al. (2014). It would increase the impact of the present study to show that these projections indeed have projection targets opposing GFP+ PBcl mice following CANE-Cre tagging.

2) The authors only use one reward, sweetened condensed milk. It would strengthen their results to examine an additional food reward, such as a solid palatable diet.

## Author Response

Significance Statement Comments for Author (Required):

N/A

Comments on the Visual Abstract for Author (Required):

N/A

Synthesis Statement for Author (Required):

Your manuscript has been read by myself and two reviewers. We all agreed that this is a well done study with appropriate controls that is a strong candidate for publication in eNeuro. However, there were a few issues that should be addressed before publication, none of which require new experimentation:

1) Reviewer #1 noted several methodological details that were insufficient and should be expanded on. The following should be addressed:

A) What was the nutritional composition and source of the condensed milk? No information is provided in the Methods as to carbohydrate/fat/protein content.

We thank reviewer #1 for this point. We have now included the nutritional composition (the percentage of carbohydrate/fat/protein) and the source of the condensed milk. In conjunction with Figure 1-1 (Extended data supporting Figure 1; see our response to point 1B and 2B), we have also included this information for chocolate Ensure, peanut butter, sucrose, and sucralose in the methods section.

B) Were the animals drinking the condensed milk? It was hard to tell if the condensed milk was in liquid or solid form from reading the manuscript.

We thank reviewer #1 for this concern. We allowed mice to drink diluted condensed milk, in liquid form, in all of our experiments. To increase the readability for general readers, we described condensed milk as a “high-caloric liquid diet” in our results section. We have also added the term “drinking” in relevant parts of our text.

If a liquid, then why didn't consumption of condensed milk also induce Fos expression in the PBsl, as water does?

We thank reviewer #1 for this critical point. In order to address this issue, we have now provided additional Fos expression experiments after consumption of another high-caloric palatable liquid (chocolate Ensure), and a palatable solid food (peanut butter), a low-caloric sweet tastant (sucrose), and non-caloric sweet tastant (sucralose).

Our results revealed that high-caloric palatable liquid/food preferentially activated PBcl, whereas low-/non-caloric sweet tastants and water (all fluids) preferentially activated PBsl. These findings compliment a recent finding from Palmiter's group (Ryan et al., Nat. Neuro 2017), in which Oxtr+ neurons residing in PBsl, the same subregion as our low-/non-caloric fluid activated neurons, expressed Fos after water or salt intake. In their paper, they showed that activation of these neurons specifically reduces fluid intake but had no effect on Chow or Ensure intake. Therefore, we believe that PBsl neurons specifically regulate low-/non-caloric fluid (water/salt/sugar) intake, but not highly caloric liquid/food intake, whereas PBcl neurons specifically regulate intake of caloric liquid/food diets.

We have cited their paper (Ryan et al., Nat. Neuro 2017) in the revised manuscript.

C) In the Methods, the authors often omit procedures and instead refer readers to previous papers (e.g. virus production procedures on p.5 or design of in situ hybridization probes on p. 7.) Please include these methods in the current Methods section.

We appreciate this concern and we have now included a more detailed description of the virus production and design of in situ hybridization probes in the methods section. We also included introductory phrases to highlight the detailed description of the RTPP and food intake assay tests.

2) Several comments were made to increase clarify and accuracy of their descriptions:

A) It seems an overreach to suggest that PBcl neurons regulate “pleasurable” satiety because only one stimulus was presented, and it happens to be a high caloric and appetitive stimulus-condensed milk. If standard chow does not activate these neurons, can they really be considered to induce a “pleasurable” satiety? Or is it more that the food is appetitive and the animals are responding to the flavor or the caloric content? More nuanced discussion of this possibility should be included.

We thank the reviewer for this constructive criticism. To address this point, we have performed additional Fos expression experiments, in which we presented separate groups of mice with 4 different palatable liquids/foods. This included consumption of another high-caloric palatable liquid (chocolate Ensure), a high-caloric palatable solid (peanut butter), a low-caloric sweet tastant (sucrose) and non-caloric sweet tastant (sucralose). We found that PBL neurons located in the same subregion, PBcl, were preferentially activated by consumption of highly caloric liquids and food, whereas consumption of low-/non-caloric sweet tastants preferentially activated PBsl neurons. Therefore, these results suggest that PBcl neurons are responding specifically to the caloric content of palatable liquids/food and not to the flavor. This may also suggest that these neurons regulate caloric-dependent satiety, which may engage in both homeostatic and hedonic neural circuitry. We have provided more discussion of this in both the results and discussion.

B) The term “Milk Activated Neurons (MANs)” seems too specific, as these neurons are likely activated by multiple stimuli (not just milk). This manuscript would make a more specific conclusion if instead of Milk Activated Neurons, they could describe specifically what these neurons respond to by examining Fos expression following different nutritional molecules (e.g. sugars, fats) or caloric parameters (e.g. refeeding after a fast). In lieu of this additional Fos staining, they could come up with a more general name for them that more accurately describes their likely function.

As per our response to point 2A, we have now provided additional Fos expression experiments after consumption of other palatable liquid/food. We have included these results in Figure 1-1 (Extended data supporting Figure 1) and discussed them in the results section. Based on the additional Fos expression experiments, PBcl neurons are indeed activated by multiple highly caloric liquids and solid food. Therefore, we have changed the term “Milk Activated Neurons (MANs)” to “Palatable-food Activated Neurons (PANs)” throughout the manuscript.

C) The title of the manuscript refers to a parabrachial “microcircuit.” It seems inappropriate to refer to the focus on PBcl neurons as a “microcircuit,” as “circuit” implies a projection between two populations of neurons and “micro” implies a circuit within a local area. Because this paper only focuses on the PBcl, the term “microcircuit” is not justified.

We thank the reviewer for this constructive criticism, and we agree with this semantic issue. We have deleted “microcircuit” and changed to “neurons” in the title and results.

3) The authors use an irreversible TeLC method to silence PBcl neurons. This is a great strategy, but it is curious that the authors only did a very short term experiment on the order of hours. It would be very interesting to know the behavior of these mice over the long-term, as PBcl silencing is long-term. Does an increase in food intake occur over several days/weeks? Does body weight continuously increase? The authors don't seem to be fully utilizing the amount of work they have put into generating these mice. More information on this should be included if possible, or a statement on why it is not possible.

We appreciate the reviewer's concern on the use of the TelC method in a short time scale. In a subset of mice, we did perform the condensed milk intake assay across multiple days prior to and after injection of TelC virus (Day 5, 6, 7, and 12). We saw an increase of condensed milk intake over days, but the effect plateaued after Day 7. Given that we are permanently silencing these neurons, one has to consider the possibility of homeostatic plasticity occurring, which is the ability of synapses to adapt to changes in their activity rate. When circuits are permanently silenced, a synaptic scaling effect may be occurring in timescales of hours to days, to compensate for downregulation of neuronal activity levels. This would result in the restoration of the circuit into its prior state of activity (Wiegert et al., Neuron 2017). Therefore, to avoid undesired side effects, while maintaining robust direct effect, we restricted fluid/food intake testing to Day 7 after TeLC injection for the remainder mice. However, we are happy to mention this increase in food consumption across days in our results section.

As per the concern of body weight gain, we did see that increased milk consumption was highly correlated with increased momentary weight gain before and after the fluid/food intake assay with condensed milk, but weight gain across the 7 days was not correlated to increased condensed milk consumption. Given that this assay is only 3 hours long, this could explain why we did not see an overall increase in body weight across days. If we had allowed the mice free access to condensed milk for 24 hours across days, we would predict an overall increase in weight gain. We have provided a statement of this in our results section.

Additional comments were made, which the reviewers and I decided were not strictly necessary for publication, so I include them here for your information, and to encourage you to include this data if you already have it or it can be easily collected.

1) A stronger attempt could be made to understand the identify of the MANs. The authors consistently refer to the fact that PVN-MC4R neurons project to the PBcl and that the neurons under present investigation are likely the same target neurons studied by Garfield et al. (2015) and Shah et al. (2014). It would increase the impact of the present study to show that these projections indeed have projection targets opposing GFP+ PBcl mice following CANE-Cre tagging.

We thank the reviewer for this concern and suggestion. Unfortunately, due to time constraints, it would be very difficult for us to perform this specific experiment to address this comment. However, we have discussed this experiment as an avenue for future directions and postulated the possible result in the discussion.

2) The authors only use one reward, sweetened condensed milk. It would strengthen their results to examine an additional food reward, such as a solid palatable diet.

We thank the reviewer for this suggestion. As per our response to point 2A and 2B., we have examined Fos expression in PBcl after consumption of peanut butter, a solid high-caloric palatable food. Consumption of peanut butter activates neurons located in PBcl similar to drinking condensed milk. We have included this data in Figure 1-1B.
